# Interaction between parasite-encoded JAB1/CSN5 and macrophage migration inhibitory factor proteins attenuates its proinflammatory function

**DOI:** 10.1038/s41598-018-28625-1

**Published:** 2018-07-06

**Authors:** Swagata Ghosh, Laura Ann Leaton, Laura Farr, Alexis Barfield, Shannon Moonah

**Affiliations:** 1 Department of Medicine, University of Virginia, Charlottesville, Virginia USA; 2 National Cancer Institute, National Institutes of Health, Bethesda, Maryland USA

## Abstract

Multiple protozoans produce homologs of the cytokine MIF which play a role in immune evasion, invasion and pathogenesis. However, how parasite-encoded MIF activity is controlled remains poorly understood. Cytokine activity can be inhibited by intracellular binding partners that are released in the extracellular space during cell death. We investigated the presence of an endogenous parasite protein that was capable of interacting and interfering with MIF activity. A screen for protein-protein interaction was performed using immunoaffinity purification of amebic cell lysate with specific anti-*Entamoeba histolytica* MIF (*Eh*MIF) antibody followed by mass spectrometry analysis, which revealed an *E*. *histolytica*-produced JAB1 protein (*Eh*JAB1) as a potential binding partner. JAB1 was found to be highly conserved in protozoans. Direct interaction between the *Eh*MIF and *Eh*JAB1 was confirmed by several independent approaches with GST pull-down, co-immunoprecipitation, and Biolayer interferometry (BLI) assays. Furthermore, the C-terminal region outside the functional JAMM deneddylase motif was required for *Eh*MIF binding, which was consistent with the top *in silico* predictions. In addition, *Eh*JAB1 binding blocked *Eh*MIF-induced IL-8 production by human epithelial cells. We report the initial characterization of a parasite-encoded JAB1 and uncover a new binding partner for a protozoan-produced MIF protein, acting as a possible negative regulator of *Eh*MIF.

## Introduction

Protozoan parasites represent a major threat to health and contribute significantly to morbidity and mortality worldwide. For example, *Entamoeba histolytica* is a protozoan parasite that causes colitis^1^. Severe forms of amebic colitis are associated with high case fatality rates ranging from 40% to 89%^2^. There is neither an effective vaccine nor have there been advancements in therapies for amebic colitis for over fifty years^3^. Therefore, there remains an ongoing need to find new drug and vaccine targets through a better understanding of parasite biology.

Macrophage migration inhibitory factor (MIF), one of the first cytokines to be discovered, is a pleiotropic inflammatory cytokine and a critical upstream mediator of innate immunity. Many of the inflammatory effects of MIF are mediated through direct binding to the CD74 cell surface receptor, causing the secretion of proinflammatory cytokines such as IL-8^4–6^. An increase in MIF expression contributes to excessive inflammation and immunopathology. Hence, MIF has been reported to have a role in the pathogenesis of several inflammatory diseases such as inflammatory bowel disease and rheumatoid arthritis^7,8^. MIF proinflammatory properties also make it a crucial mediator in the immune response against a wide range of pathogens^9–11^.

Counterintuitively, MIF homologs have been characterized in several pathogenic protozoans including *Entamoeba*, *Plasmodium*, *Toxoplasma*, and *Leishmania*. These protozoan MIF homologs have demonstrated similar proinflammatory activities to that of human MIF, and play a role in immune evasion, invasion and pathogenesis^12–19^. Despite the growing literature on protozoan-encoded MIF proinflammatory activity, very little is known about how it is regulated. Here, we uncover a parasite-encoded JAB1 (c-Jun activation domain binding protein 1), which is highly conserved throughout protozoan parasites, as a novel binding partner and potential regulator of the MIF homolog of *Entamoeba histolytica*.

## Results

### Identification and characterization of a parasite-produced JAB1

**Figure 1:**
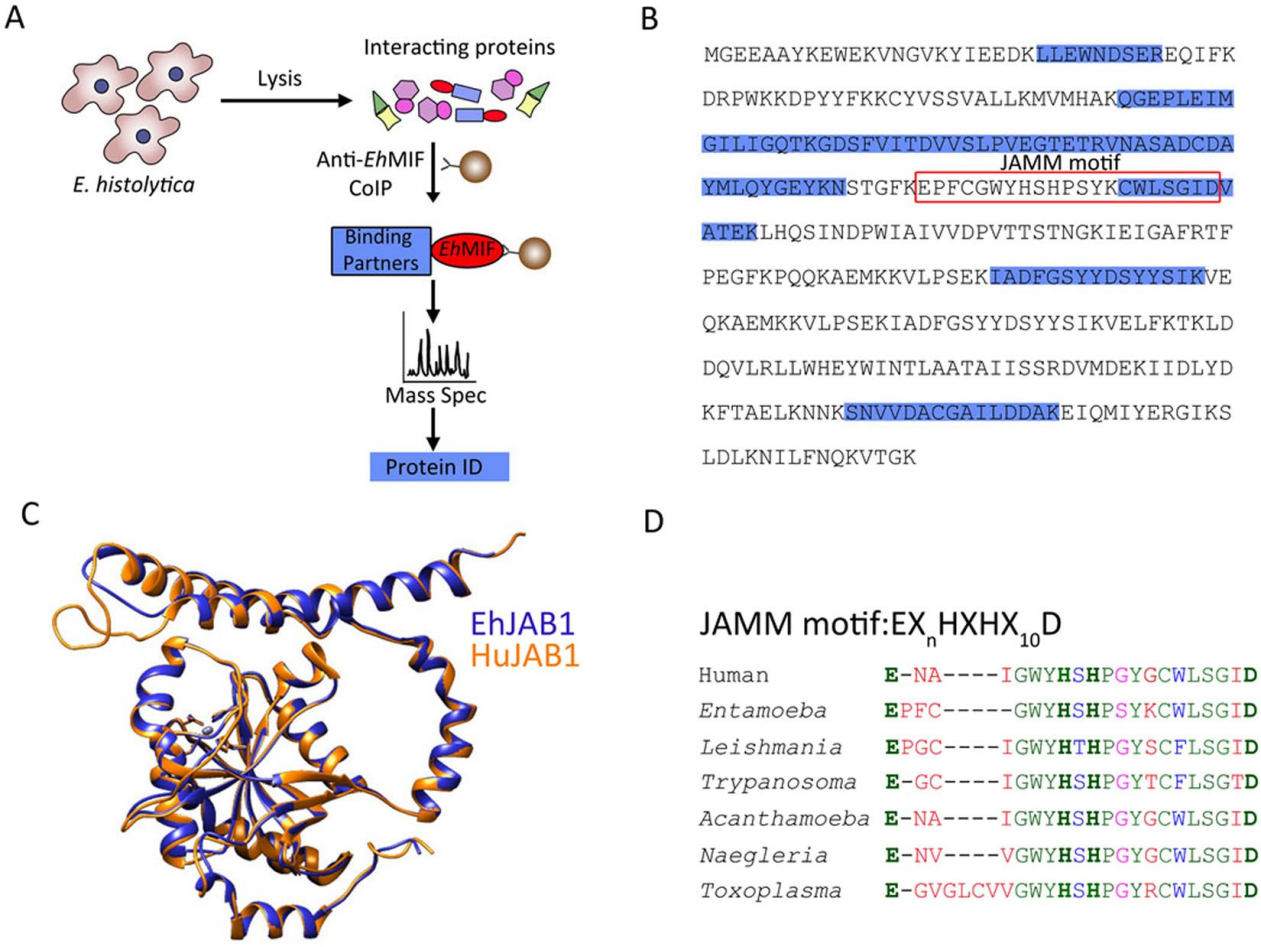
Characterization of *Eh*JAB1. (**A**) Schematic of procedure for identifying novel protein-protein interaction with co-immunoprecipitation followed by mass spectrometric analysis. (**B**) *E*. *histolytica* JAB1 (*Eh*JAB1) amino acid sequence. Peptides unique to the *Eh*JAB1 protein identified by mass spectrometry are highlighted (blue). JAMM motif (EX_n_HXHX_10_D) shown in red box. (**C**) Structural homology between Human JAB1 (HuJAB1) and *Eh*JAB1. HuJAB1 (orange) was superimposed with the predicted structure of *Eh*JAB1 (blue). (**D**) Multiple sequence alignment of the conserved JAMM motif of JAB1 from pathogenic parasites. Identical (green), conserved (blue), semi-conserved (pink), and non-conserved residues (red).

Co-immunoprecipitation (co-IP) of proteins followed by mass spectrometric identification is a standard approach for identifying novel protein-protein interactions^20,21^. Parasite cell lysates were incubated with a specific antibody against *Entamoeba histolytica* MIF (*Eh*MIF) or IgG control antibody. The immunoprecipitates were subjected to mass spectrometry analysis. An *Entamoeba histolytica*-encoded JAB1 (*Eh*JAB1, *EH*I_050500) was identified as a potential *Eh*MIF binding partner (Fig. 1A,B). While a number of putative interacting proteins were co-precipitated with anti-*Eh*MIF antibody, *Eh*JAB1 was selected for further analysis given: (i) *Eh*JAB1 was detected in the anti-*Eh*MIF co-IP sample and not detected in the control IP; (ii) *Eh*JAB1 was among the highest percent coverage (Supp. Fig. S1); and (iii) previous report of the intracellular interaction between human MIF and human JAB1 (HuJAB1)^22–24^. *Eh*JAB1 gene consists of 957 base pairs with no intron and a GC content of 30%. The gene encodes for a protein of a predicted molecular weight of 36.5 kDa.*Eh*JAB1 has 39% identity and 61% similarity with HuJAB1 and appears structurally conserved (Figs 1C and S2). JAB1 is conserved in multiple parasites that cause disease in humans, all containing the MPN domain with the JAMM (JAB1/MPN/Mov34 metalloenzyme) motif (Fig. 1D). JAMM motif is a metalloprotease motif, consisting of the amino acid sequence EX_n_HXHX_10_D, present in the deubiquitinating enzymes RPN11 and JAB1^25^. RPN11 was also found is these parasites (Supp. Fig. S3).

### *Eh*MIF directly interacts with *Eh*JAB1

**Figure 2:**
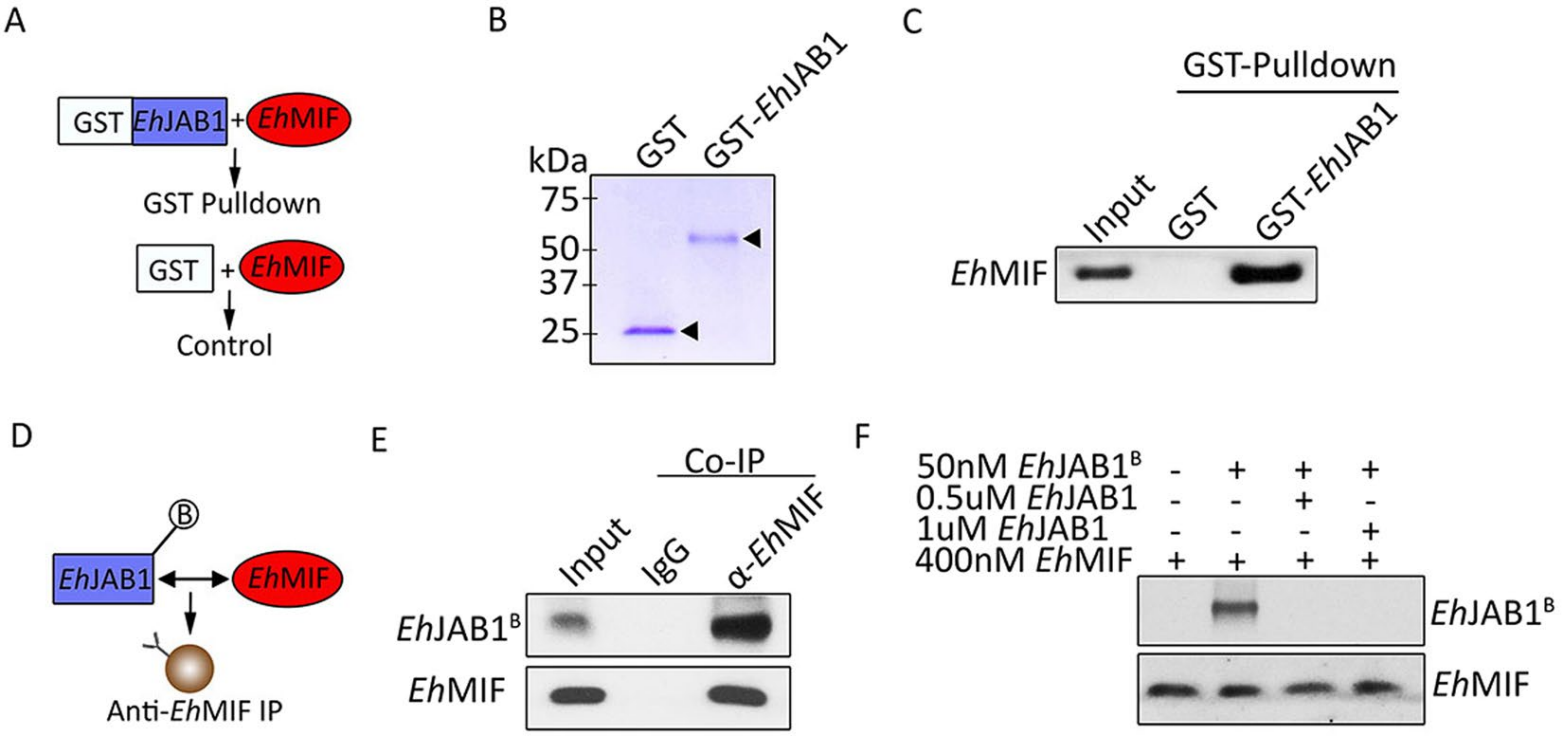
Direct interaction between *Eh*MIF and *Eh*JAB1. (**A**) Schematic of GST pull-down assay. (**B**) Purified recombinant GST and GST-*Eh*JAB1 proteins (arrowhead) used in the GST pull-down assay were separated by SDS/PAGE and stained with Coomassie Blue. (**C**) Interaction between *Eh*MIF and *Eh*JAB1 by GST pull-down assay. GST-*Eh*JAB1 and GST control were mixed with *Eh*MIF. Input (10%) and pull-down material were separated by SDS/PAGE, and *Eh*MIF was detected by immunoblot analysis using anti-*Eh*MIF antibody. (**D**) Schematic diagram of co-immunoprecipitation assay. (**E**,**F**) Co-immunoprecipitation of *Eh*MIF and biotinylated *Eh*JAB1 (*Eh*JAB1^B^) mixture using specific anti-*Eh*MIF antibody bound beads. *Eh*JAB1^B^ was detected by immunoblot analysis using goat anti-biotin HRP conjugated antibody.

We investigated whether the interaction between *Eh*MIF and *Eh*JAB1 was direct or indirect. First, we performed GST-pulldown assays (Fig. 2A). *Eh*MIF and *Eh*JAB1 fused to GST were expressed and purified (Fig. 2B). GST-*Eh*JAB1 immobilized on magnetic beads was incubated with *Eh*MIF. GST alone immobilized on magnetic beads and incubated with the same concentration of *Eh*MIF was used as control. As shown in Fig. 2C, the GST-*Eh*JAB1 fusion protein readily pulled down *Eh*MIF relative to the GST control, demonstrating direct interaction. In an independent approach, we used co-immunoprecipitation experiments to further verify direct interaction. *Eh*JAB1 immunoprecipitated with anti-*Eh*MIF antibody, but not with the IgG antibody control (Fig. 2D–F). Together, these findings suggest a direct binding between *Eh*MIF and *Eh*JAB1 proteins.

### High-affinity binding between *Eh*MIF and *Eh*JAB1 proteins

**Figure 3:**
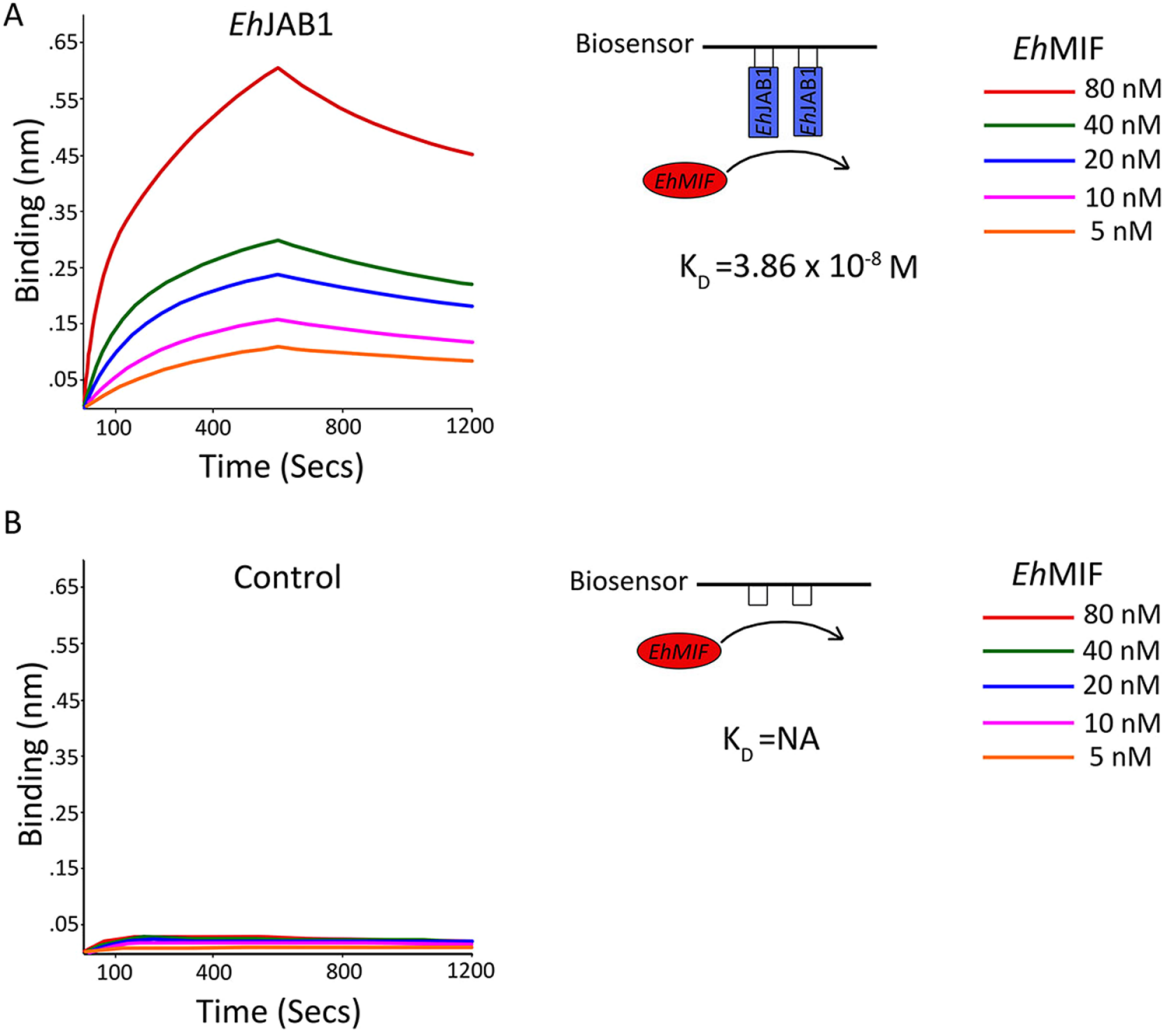
Characterization of *Eh*MIF binding to *Eh*JAB1 using the Biolayer interferometry. (**A**) Representative binding and dissociation curves for *Eh*MIF binding to *Eh*JAB1. Anti-GST antibody-coated biosensors were loaded with GST-*Eh*JAB1. Sensors were placed into solutions with *Eh*MIF, concentrations range from 5 to 80 nM (association analysis: 0 to 600 secs). Subsequently, the sensors were transferred to buffer without *Eh*MIF for dissociation analysis (from 600 to 1200 secs). Analysis revealed a dissociation constant of K_D_ of 3.86 × 10^−8^ M. (**B**) Biosensors loaded with GST only were used as controls.

Biolayer interferometry (BLI) is a useful technique for measuring interactions between proteins in real time^26^. We determined the equilibrium dissociation constant for *Eh*MIF binding to *Eh*JAB1 using BLI. GST-tagged *Eh*JAB1 was coupled to the surface of anti-GST antibody coated BLI sensors, followed by binding measurements in different concentrations of *Eh*MIF. Analysis revealed a dissociation constant of K_D_ of 3.86 × 10^−8^ M (Fig. 3A). GST alone coupled to anti-GST antibody coated BLI sensors was used as control. BLI measurements demonstrate that *Eh*MIF did not bind to the GST control, K_D_ not applicable (Fig. 3B). These findings suggest *Eh*MIF binds to *Eh*JAB1 within the range considered biologically relevant^27,28^.

### *Eh*JAB1 interacts with *Eh*MIF via its C-terminal domain

**Figure 4:**
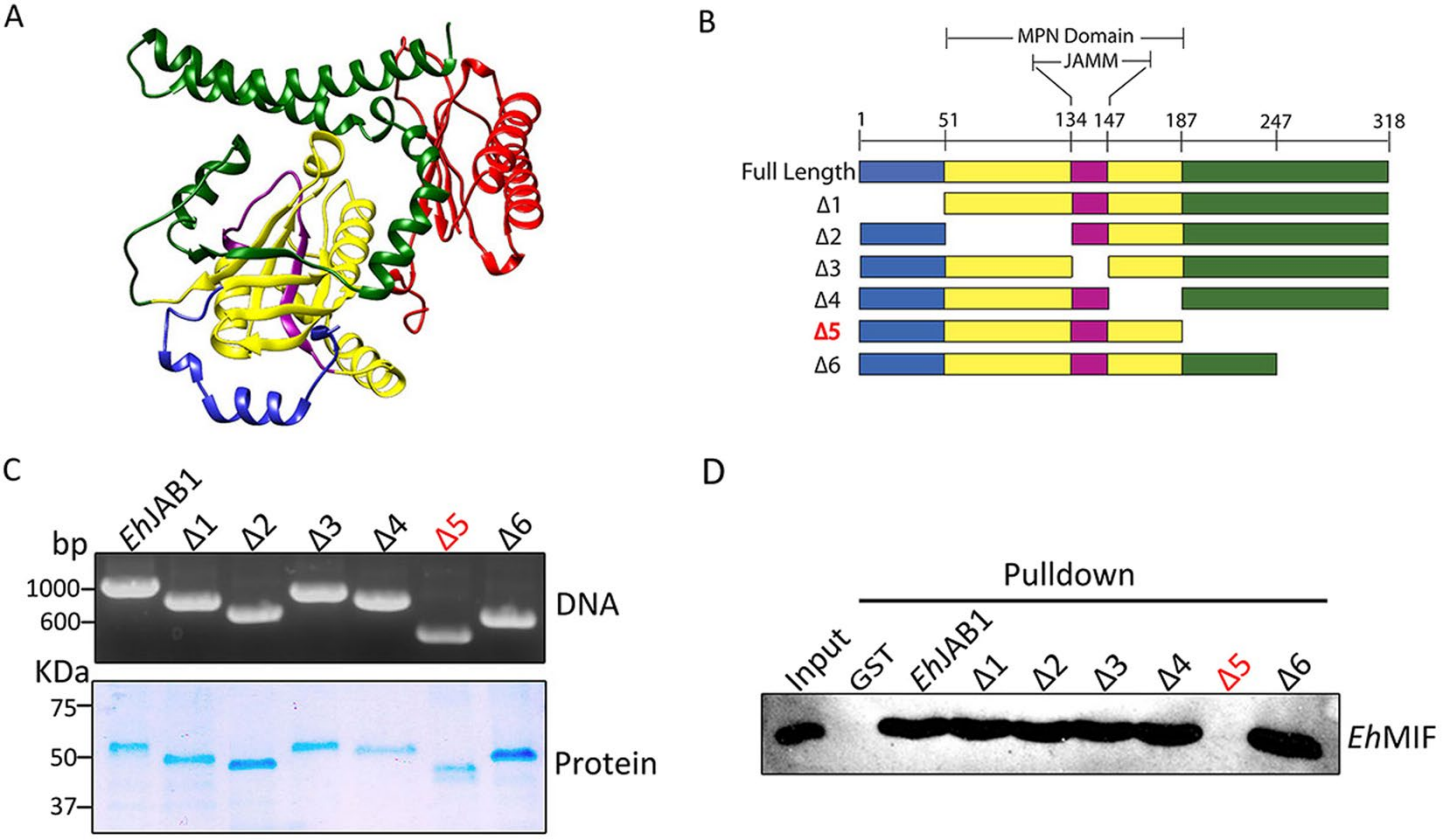
Mapping the *Eh*MIF-interacting domain of *Eh*JAB1. (**A**) Predicted interaction between *Eh*MIF and *Eh*JAB1. *Eh*MIF (red) and *Eh*JAB1 N*-*terminal (blue), MPN domain (yellow), JAMM motif (purple) and C-terminal (green). (**B**) Schematic representations of full-length and deletion mutants of *Eh*JAB1. Δ 1 (deletion of 3–50 aa), Δ 2 (deletion of 51–133 aa), Δ 3 (deletion of 134–147 aa), Δ 4 (deletion of 148–187 aa), Δ 5 (deletion of 187–318 aa), Δ 6 (deletion of 247–318 aa). (**C**) PCR amplicons and protein expression of GST-tagged full-length *Eh*JAB1 and GST*-*tagged deletion mutants of *Eh*JAB1. (**D**) Interactions of *Eh*MIF with deletion mutants were analyzed by immunoblotting.

We investigated the binding domain of *Eh*JAB1 responsible for interacting with *Eh*MIF. First, an *in silico* approach using pyDockWEB was applied to assess the binding of *Eh*JAB1 to *Eh*MIF. pyDockWEB allows the best rigid-body docking orientations generated by pyDock scoring function, which consists of electrostatics, desolvation energy and limited van der Waals contribution^29^. The C-terminal domain of *Eh*JAB1 was revealed to be the *Eh*MIF binding region in all top ten predictions (Figs 4A and S6). Next, we further examine the region of *Eh*JAB1 that is responsible for the formation of the complex with *Eh*MIF using deletion mutant analysis. GST-tagged full-length *Eh*JAB1 and various GST*-*tagged deletion mutants of *Eh*JAB1, with either N-terminal, MPN, JAMM or C-terminal domain deletion (Fig. 4B,C), were immobilized on magnetic beads and incubated with *Eh*MIF protein. *Eh*MIF specifically interacted with GST–*Eh*JAB1, except for the mutant lacking the C-terminal domain. Interaction was regained when the 187–246 amino acid sequence of the C-terminal was restored (Fig. 4D). These results indicate binding of *Eh*JAB1 to *Eh*MIF is mediated through the C-terminal region outside of the MPN domain.

### *Eh*JAB1 inhibits *Eh*MIF interaction with the CD74 receptor and *Eh*MIF-induced cytokine production

**Figure 5:**
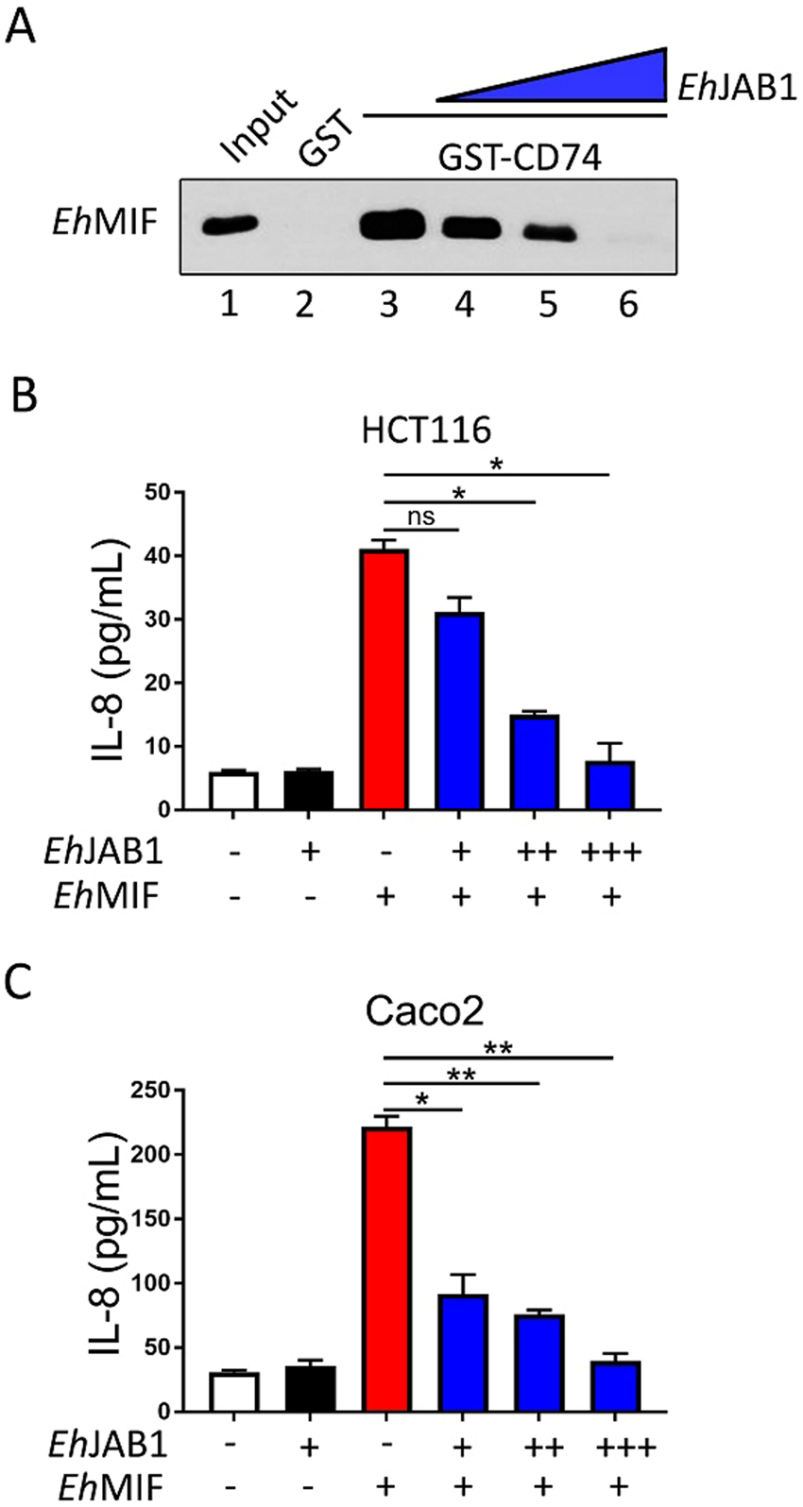
Effect of *Eh*JAB1 binding on *Eh*MIF activity. (**A**) *Eh*JAB1 inhibits *Eh*MIF interaction with the human MIF receptor CD74 by GST pull-down competition assay. Input (lane 1) and GST control (lane 2). *Eh*MIF was preincubated with or without *Eh*JAB1 at increasing doses before mixing with GST-CD74 (lanes 3–6). (**B**,**C**) *Eh*JAB1 inhibits *Eh*MIF-induced IL-8 production in a dose-dependent manner. Human colonic epithelial cells HCT116 and Caco2 were incubated with *Eh*MIF with or without *Eh*JAB1 at increasing doses. Culture supernatants were collected after 8 h and IL-8 was quantified by ELISA. Data represent mean and SD of triplicates from 1 experiment and are representative of 3 independent experiments. *P < 0.01; **P < 0.001. ns, not significant.

CD74 is a cell surface receptor for human MIF, which mediates many of its inflammatory effects^30^. Several parasite MIF homologs, including *Eh*MIF, were previously shown to interact with the CD74 receptor^13,17,18,31^. Here, we were able to reproduce this finding and found that *Eh*MIF-CD74 interaction was blocked by preincubating *Eh*MIF with *Eh*JAB1 (Fig. 5A). The epithelial surfaces of the skin, nasal, intestinal, respiratory, and genitourinary tracts are the first points of contact for many protozoans. Epithelial cells express CD74 and are a rich source of IL-8^32^. Stimulation of IL-8 production has been the most reproducible activity of protozoan MIF homologs. *Eh*MIF was recently shown to stimulate IL-8 secretion from human intestinal epithelial cells^19^. IL-8 is a potent neutrophil chemoattractant that contributes to inflammation in various infectious and inflammatory diseases. We proceeded to examine if *Eh*JAB1 binding to *Eh*MIF attenuates its proinflammatory function using two different colonic epithelial cell lines. *Eh*JAB1 blocked *Eh*MIF-induced IL-8 secretion by HCT116 and Caco2 colonic cells (Fig. 5B,C). These data indicate that when bound to *Eh*JAB1, *Eh*MIF is no longer capable of carrying out its inflammatory functions.

## Discussion

Parasites can exploit the host inflammatory response to promote tissue invasion^1^. *Eh*MIF-stimulated human intestinal epithelial cells secrete IL-8, a potent promoter of inflammation^19^. One of the downstream effects of *E*. *histolytica* MIF-induced inflammation is an increase in matrix metalloproteinases (MMPs) production, which was shown recently to promote *E*. *histolytica* tissue invasion in human colon^19,33^. That said, parasites are likely to have developed mechanisms to regulate their MIF actions especially in situations where they fail to evade the host inflammatory response induced by producing such a molecule.

Identifying a binding partner that inhibits MIF function provides insight into how MIF’s actions are regulated^27^. In this study, a proteomics approach was used to identify parasite-encoded protein interaction partners of a homolog of MIF from *Entameoba histolytica*. We found that *Eh*JAB1 protein interacts with *Eh*MIF. This interaction was validated by multiple independent approaches with GST-pulldowns, co-immunoprecipitation, *in silico* experiments and Biolayer interferometry.

JAB1, also known as COP9 signalosome subunit 5 (CSN5), is well characterized in non-parasitic eukaryotes^34^. It constitutes the catalytic center of the large multi-protein COP9 signalosome complex, since it harbors the JAMM/MPN^+^ metalloprotease motif^35–37^. It carries out a Zn dependent reaction called deneddylation, equivalent to de-ubiquitylation, where it removes a ubiquitin (Ub) like protein Nedd8 from the Cullin-RING E3 Ub-ligases (CRL)^35,38,39^. Regulation of CRLs, such as the Skp/Cullin/F-box (SCF) complex, by CSN mediated deneddylation is critical for proper cell division, cell cycle control and DNA damage response^40,41^. Here, we report the initial characterization of a parasite-encoded JAB1. Given parasites including *E*. *histolytica* express cullins and Nedd8 proteins^42–45^, it is plausible that JAB1 in parasites regulate cullins by deneddylation and modulate ubiquitin proteasomal system (UPS) activity, however, further studies to confirm this are needed. Surprisingly, we did not find a JAB1/CSN5 homolog in *Plasmodium* genome. However, the incomplete genome assembly and annotation in *Plasmodium spp*. could limit our *in-silico* analysis and explain why JAB1 was not identified in *Plasmodium spp*.

JAB1 function is not limited to CSN dependent deneddylation. JAB1 is also stable and functional in a free, monomeric, CSN independent form^46–48^. JAB1 monomers are catalytically inactive, but bind to certain proteins and alter their activity^34,48^. We showed that the JAMM catalytic motif of *Eh*JAB1 was not required for its interaction with *Eh*MIF, which is consistent with the previous report on mammalian proteins^23^. The dissociation constant for *Eh*JAB1-*Eh*MIF complex was well within the range considered physiologically relevant, suggesting a biologically significant interaction^27,28^.

Intracellular molecules released from damaged cells can have profound effects on the immune response. There is growing evidence that not all of these immunomodulatory molecules are pro-inflammatory, and interestingly, some have immunosuppressive or regulatory effects. These anti-inflammatory actions occur either directly or indirectly. For example, in the case of human MIF, an endogenous binding partner that is released in the extracellular space during cell death interacts and negatively interferes with MIF activity^27,49–51^. JAB1 is an intracellular protein^22^, which is consistent with our mass spectrometric analysis of the parasite cytosolic fraction; while MIF homologs, such as *Eh*MIF are secreted proteins^18,19^. *E*. *histolytica* parasite has developed a number of mechanisms to evade the host immune response^1,52^. However, amebic parasites could become damaged if they fail to evade the inflammatory response triggered by *Eh*MIF. It would be reasonable to speculate that the free monomeric *Eh*JAB1 released from injured cells into the extracellular environment would then form complexes with *Eh*MIF, preventing interaction with the host receptor CD74 and reducing *Eh*MIF-induced inflammation, as a negative feedback mechanism. In addition, given the structural and functional similarity between human and *E*. *histolytica* MIF and JAB1 proteins, we do postulate that *Eh*JAB1 could also interact with human MIF. It would be interesting to determine whether an *Eh*JAB1/human MIF interaction functions to evade the host immune response in future studies.

In conclusion, our data suggest that *E*. *histolytica* homolog of JAB1 interacts with the cytokine *Eh*MIF and negatively regulates its pro-inflammatory function. Our study also generates the hypothesis that targeting *Eh*MIF and its interaction with *Eh*JAB1 may disrupt the parasite’s ability to exploit the host immune response and ultimately serve as a therapeutic target against this devastating parasitic disease.

## Methods

### Plasmids, cloning and PCR

The *Eh*JAB1 gene, codon optimized for expression in *E*.*coli* BL21(DE3) cells, was cloned within pDEST15 vector. Deletion constructs were prepared by inverse PCR on the pDEST15-full length JAB1 clone using the primers listed in the Supp. Fig. S4. A schematic of the mutation strategy is provided in the Supp. Fig. S5. Clones were screened by PCR across the gene boundaries within the vector followed by confirmation with sequencing. The *Eh*JAB1 gene was amplified from the pDEST15 vector with primers carrying 5′BamHI and 3′XhoI sites and sub-cloned within BamHI and XhoI sites of pGEX-4T1 vector to utilize the thrombin site for cleaving off the GST tag from the GST-*Eh*JAB1 protein. Also, the previously described codon optimized *Eh*MIF gene cloned within pJexpress414 vector (DNA2.0)^17^ was used in this study.

### Protein expression and purification

Protein expression of the recombinant *Eh*MIF and *Eh*JAB1was done following the previously described protocol^17^ except that the induction with isopropyl β- D –thiogalactoside (IPTG) was done for 18 hours at 15 °C. Cells were pelleted and lysed in CelLytic^TM^ B Cell Lysis Reagent (Sigma) at room temperature for 15 minutes and lysate was collected following 30 min spin at maximum speed at 4 °C. The purification of GST-fusion protein and His-tagged fusion protein was done as previously described^17^ using glutathione-sepharose (GE-Healthcare) and Ni-NTA agarose beads (Qiagen) respectively. Polymyxin B (Sigma) was used in the purification procedures for the removal of endotoxin. On-column cleavage of the GST-*Eh*JAB1 protein was done with the Thrombin Cleavage Capture Kit (Millipore) that utilizes biotinylated thrombin for its streptavidin agarose based removal.

### GST-Pulldown Assay

GST pull down assays were done using the MagneGST^TM^ Protein Purification System kit (Promega). Bacterial lysate expressing GST fused- full length or deletion mutant *Eh*JAB1 protein, diluted in MagneGST binding/wash buffer to a concentration 500 ng/ml in 500 µl, was mixed with 25 µl magnetic beads and was incubated overnight at 4 °C with rotation. Beads were washed 3 times with 500 µl binding/wash buffer followed by incubation with 1 µg purified *Eh*MIF at 4 °C for overnight. The GST-CD74 pulldown assays were performed as previously described^17,31^. Briefly, *Eh*MIF protein was pre-incubated with or without 1X, 2X or 5X concentration of *Eh*JAB1 protein for 30 minutes in 500 µl binding/wash buffer prior to incubation with the GST-CD74 bound magnetic beads at 4 °C for overnight. The bound complexes were eluted in 40 µl elution buffer after washing the beads 5 times with 500 µl binding/wash buffer. In a control experiment equivalent amount of GST protein was used in place of GST-*Eh*JAB1 or GST-CD74 proteins. Twenty five percent of the eluted samples were assayed by immunoblots.

### Immunoprecipitation

Approximately 1.25 × 10^7^ trophozoites of *E*. *histolytica*, grown at 37 °C in TYI-S-33 medium, were harvested in 3 ml non-denaturing lysis buffer (20 mM Tris HCl pH 8, 137 mM NaCl, 1% TritonX-100, 2 mM EDTA, 1x Protease inhibitor freshly added). Lysis was done with sonication followed by centrifugation at high speed for 10 minutes at 4 °C. Immunoprecipitations were performed with anti-*Eh*MIF rabbit serum^17^ and rabbit IgG (Santa Cruz Biotechnology). For each, 100 µl Dynabeads^TM^ Protein A (Invitrogen) was chemically conjugated with 10 µg antibodies using 5 mM Bis[Sulfosuccinimidyl] suberate or BS^3^ (Thermo Scientific) following the manufacturer’s manual. Antibody conjugated beads were added to 500 µl amebic lysate and incubated overnight at 4 °C with rotation. Following binding, beads were washed 5 times with 300 µl wash buffer (10 mM Tris HCl pH 7.4, 150 mM NaCl, 1% TritonX-100, 1 mM EDTA, 1x Protease inhibitor freshly added). The bound complexes were eluted in 40 µl 1x Laemmli sample buffer (Bio-Rad) by boiling. Co-precipitated endogenous proteins were analyzed by Mass-Spectrometry. For direct binding experiment, tag free *Eh*JAB1protein was biotinylated using the EZ-Link^R^ Sulfo-NHS-LC-Biotinylation kit (Thermo Scientific). Immunoprecipitation was performed with 50 µl dynabeads bound to 10 µg anti-*Eh*MIF rabbit serum or rabbit IgG. For each set, 1.5 µg of *Eh*MIF mixed with 500 ng biotinylated *Eh*JAB1protein was incubated with the antibody coated beads in 300 µl binding buffer (as described above) for overnight at 4 °C with rotation. Wash and elution of the bound complexes were done the same way as above. The immunoprecipitation was repeated in the presence of 5 µg and 10 µg non-biotinylated tag free *Eh*JAB1 protein. Twenty-five percent of the eluted samples were assayed by immunoblots.

### Mass Spectrometry

The entire eluted fraction from anti-*Eh*MIF and rabbit IgG immunoprecipitations were subjected to electrophoresis on SDS polyacrylamide gel. Proteins were separated via SDS-PAGE for a length of 1 cm^53^. About 1 cm × 1 cm section of the gel, spanning all of the resolved proteins in each well, were excised. The gel samples were submitted to the W. M. Keck Biomedical Mass Spectrometry Laboratory for mass spectrometry analysis.

### Immunoblotting

Protein samples obtained from GST-pulldown and immunoprecipitation experiments were resolved by SDS-PAGE followed by transfer onto polyvinylidene difluoride membranes (Millipore). The membranes were incubated overnight with primary antibodies at 4 °C. For *Eh*MIF protein detection, rabbit anti-*Eh*MIF antibody was used followed by anti-rabbit IgG HRP conjugate (Sigma) secondary antibody. For biotinylated *Eh*JAB1, goat anti-biotin HRP conjugated antibody (Cell Signaling Technology) was used. Enhanced chemiluminescence (Thermo scientific) based substrates were used to detect antibody conjugated peroxidase activity.

### IL8 secretion assay by ELISA

The human colonic epithelial cells (HCT116 & Caco2, American Type Culture Collection) with densities 10^6^ cells/ml were cultured in 48 well plate (Corning) with 100 µL complete media (Dulbecco’s Modified Eagle Medium, Gibco) for 12 hours followed by gentle washing and incubation with 100 µl serum free media for 12 hours. After washing the plates with 200 µl media, cells were treated with 0.5 µg/ml *Eh*MIF in presence or absence of 0.5, 1 and 2.5 µg/ml *Eh*JAB1 protein for 8 hours. IL-8 in cell culture supernatant was measured by enzyme linked immunosorbent assay (ELISA, eBioscience).

### Binding kinetics using BLI assay

The binding affinities between GST-*Eh*JAB1 and *Eh*MIF proteins were measured using the Blitz System (Octet^R^ Red 96 system, ForteBio). Briefly, Anti-GST Dip and Read^TM^ Biosensors (ForteBio) were hydrated for 10 minutes in the sample dilution buffer (1x DPBS, 0.1% BSA, 0.02% Tween 20) followed by 3 cycles of priming and neutralization, 20 seconds each, in regeneration buffer (10 mM Glycine, pH 1.7) and the sample dilution buffer, respectively. Next, baseline stabilization of the primed biosensors was done in the dilution buffer for 5 minutes. Then, 10 µg GST-JAB1 or GST proteins diluted in sample dilution buffer was loaded onto the biosensors for 5 minutes. After washing the loaded biosensors in sample dilution buffer for 5 minutes, they were exposed for 10 minutes to 5, 2-fold dilution series of *Eh*MIF protein starting at 80 nM concentration. Post-binding dissociation of *Eh*MIF was done for 10 minutes in the sample dilution buffer. Binding affinities (K_D_) were calculated using the Blitz system software (ForteBio).

### Bioinformatics

Orthologues of JAMM/MPN^+^ motif of the *Eh*JAB1 protein from different protozoan parasites were aligned by Multiple Sequence Comparison by Log Expectation (MUSCLE) software^54^. 3D structure of *Eh*JAB1 was constructed by Protein Homology/Analogy Recognition Engine v 2.0 (PHYRE^2^)^55^. The predicted *Eh*JAB1 structure was then compared with that of the Human JAB1 protein using the UCSF Chimera software v. 1.10.2. The protein-protein interaction between *Eh*JAB1 and *Eh*MIF was examined and potential docking interfaces were predicted using pyDockWeb that applies a rigid body protein-protein docking prediction model by electrostatic and desolvation scoring^29^. The predictions were viewed using the UCSF Chimera software v. 1.10.2.

### Statistical Tests

Statistical differences were determined using ANOVA followed by Dunnett’s post-hoc test. A p value less than 0.05 was considered statistically significant.

## Electronic supplementary material

Supplementary Information
