# Supplementary material for: Interaction between parasite-encoded JAB1/CSN5 and macrophage migration inhibitory factor proteins attenuates its proinflammatory function
Source: Sci Rep. 2018 Jul 6;8:10241. doi: 10.1038/s41598-018-28625-1 (PMC6035221; doi:10.1038/s41598-018-28625-1)

Supplementary Information

Interaction between parasite-encoded JAB1/CSN5 and macrophage migration inhibitory factor proteins attenuates its proinflammatory function

Swagata Ghosh[1], Laura Ann Leaton[1], Laura Farr[1], Alexis Barfield[2] and Shannon Moonah[1]*
[1]Department of Medicine, University of Virginia, Charlottesville, Virginia, USA
[2]National Cancer Institute, National Institutes of Health, Bethesda, Maryland, USA
*correspondence author sm5fe@virginia.edu

Protein	Gene ID	Protein Identification Probability	Percent Coverage
Ribosomal Proteins*	EHI_073600	100%	62%
Macrophage Migration Inhibitory Factor (MIF)	EHI_092370	100%	54%
Histone#	EHI_182990	100%	41%
Copine	EHI_140260	100%	38%
Ribonucleoprotein complex subunit 4	EHI_115300	100%	36%
JAB1/CSN5	EHI_050500	100%	33%
Molybdenum cofactor sulfurase	EHI_027700	100%	33%
Sulfate adenylyltransferase	EHI_197160	100%	32%
Inorganic pyrophosphatase	EHI_124880	100%	32%
Skp1	EHI_066770	100%	31%

Figure. S1 Top proteins detected by mass spectrometry. *Represents 40S ribosomal protein S15a. #Represents histone H2B.

**Name**	**ID% w/ Hu-JAB1**	**Sim% w/ Hu-JAB1**	**Length (aa)**	**MW(Da)**	**IP**
Human	100%	100%	334	37.5	6.1
Entamoeba	39%	61%	318	36.5	5.35
Toxoplasma	41%	54%	489	53.6	5.61
Trypanosoma	35%	53%	398	44.7	6.19
Leishmania	34%	53%	473	51.4	7.72
Acanthamoeba	71%	79%	311	34.8	5.87
Naegleria	75%	85%	310	34.8	6.56

Figure. S2 Human JAB1/CSN5 (ENSG00000121022) orthologues in parasites: *Entamoeba histolytica* (EHI_050500), *Toxoplasma gondii* (TGARI_308590), *Trypanosoma Cruzi* (TcCLB.507083.60), *Leishmania donovani* (LBRM2903_160015100), *Acanthamoeba castellanii* (ACA1_074760), *Naegleria fowleri* (NF0060740). Listed are the level of their sequence identity and similarity with the human protein, length of amino acid sequence, molecular weight and isoelectric point.

Human	EMVVGWYHSHPGFGCWLSGVD
Entamoeba	ESIVGWYHSHPGFGCWLSSID
Trypanosoma	EKVVGWYHSHPGFGCWLSGED
Leishmania	ENVVGWYHSHPGFGCWLSSED
Toxoplasma	EMVVGWYHSHPGFGCWFSGTD
Acanthamoeba	EMVVGWYHSHPGFGCWLSGVD
Naegleria	EMVVGWYHSHPGFGCWLSSVD

Figure.S3 Alignment of the conserved JAMM motif of RPN11 from human (ENSG00000115233) and pathogenic parasites: *Entamoeba histolytica* (EHI_164750), *Toxoplasma gondii* (TGARI_269840), *Trypanosoma Cruzi* (TcCLB.503697.9), *Leishmania donovani* (LBRM2903_200012600), *Acanthamoeba castellanii* (ACA1_073080), *Naegleria fowleri* (NF0076640). Identical (green), conserved (blue), semi-conserved (pink), and non-conserved residues (red).

#	Sequence	Use
1	5’ ACCCATGAAGCCTGCTTTTTTGTACAAA 3’	Δ3-50 *Eh*JAB1 (Δ1)
2	5’ TGCTATGTTAGCAGCGTGGCA	
3	5’ CTTTTTGAAATAATACGGATCTTTTTTCCACGG 3’	Δ51-133 *Eh*Jab1 (Δ2)
4	5’ CATAGCCATCCGAGCTATAAATGTTGG 3’	
5	5’ ATACCAACCGCAAAACGGTTCTTTAAAACC 3’	Δ134-147 *Eh*Jab1 (Δ3)
6	5’ GTTGCAACCGAAAAACTGCATCAGAGC 3’	
7	5’ ATCAATACCGCTCAGCCAACATTTATAGCT 3’	Δ148-187 *Eh*Jab1 (Δ4)
8	5’ GGTTTTAAACCGCAGCAGAAAGCC 3’	
9	5’ ATTCCGGAAAGGTACGAAAGGC 3’	Δ187-318 *Eh*Jab1 (Δ5)
10	5’ AAGACCCAGCTTTCTTGTACAAAGTG 3’	
11	5’ ATGCCAGGGTATTAATCCAATATTCATGC3’	Δ247-318 *Eh*Jab1 (Δ6)
12	5’ AAGACCCAGCTTTCTTGTACAAAGTG 3’	
13	5’ AACAAGTTTGTACAAAAAAGCAGGCTTC 3’	pDEST15-*Eh*JAB1 deletion screen
14	5’ CACTTTGTACAAGAAAGCTGGGTCTTA 3’	
15	5’ AAAAAAGGATCCATGGGTGAAGAGGCAGCATATA 3’	5’BamH1 - *Eh*JAB1- 3’ Xho1
16	5’ AAAAAACTCGAGTTATTTGCCGGTCACTTTCTGA 3’	

Figure. S4 Primers used for making the *Eh*JAB1 deletion constructs by inverse PCR (#1-12), screening of deletion constructs (#13-14) and sub-cloning of *Eh*JAB1 in pGEX-4T1 vector (#15-16) are described.

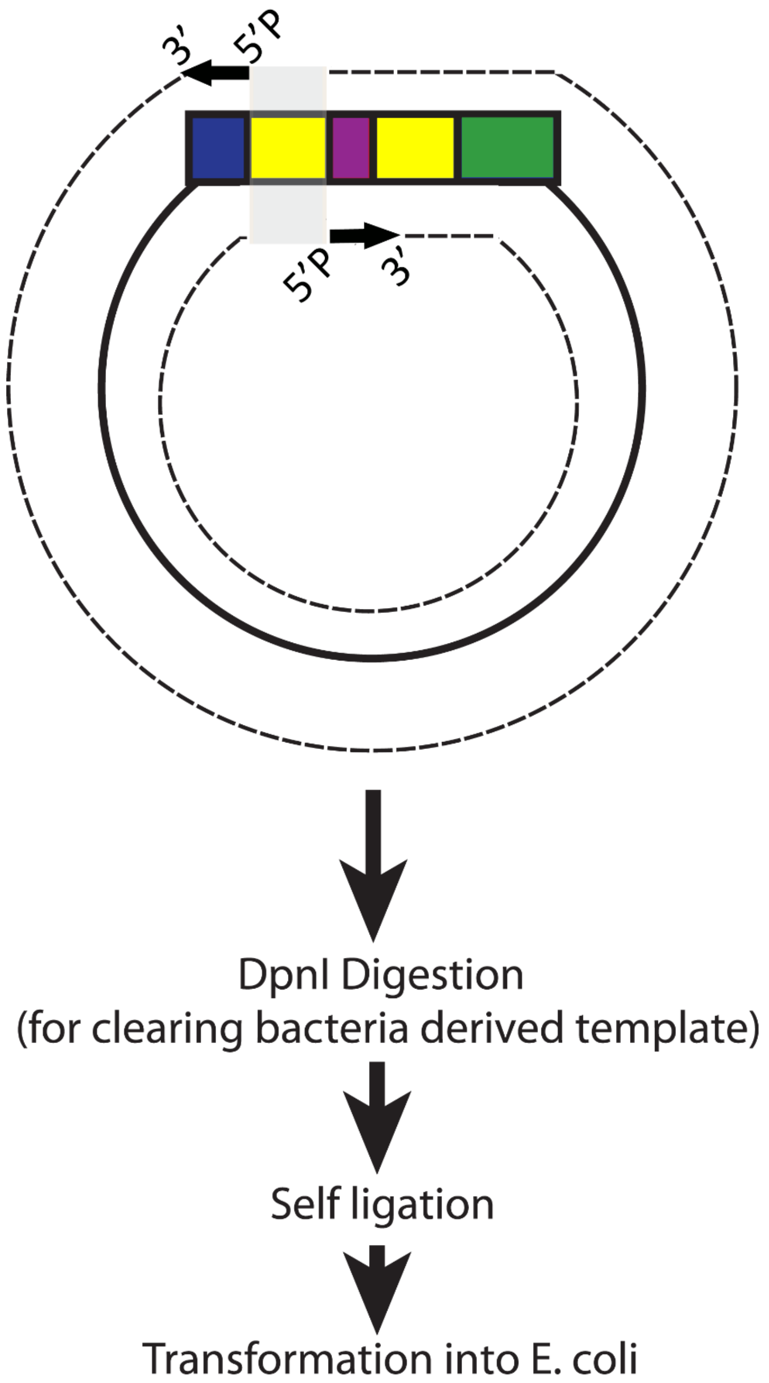

Figure.S5 Schematic of the strategy and steps for making the deletion mutants of *Eh*JAB1. Inverse PCR done with 5’phosphorylated primers.

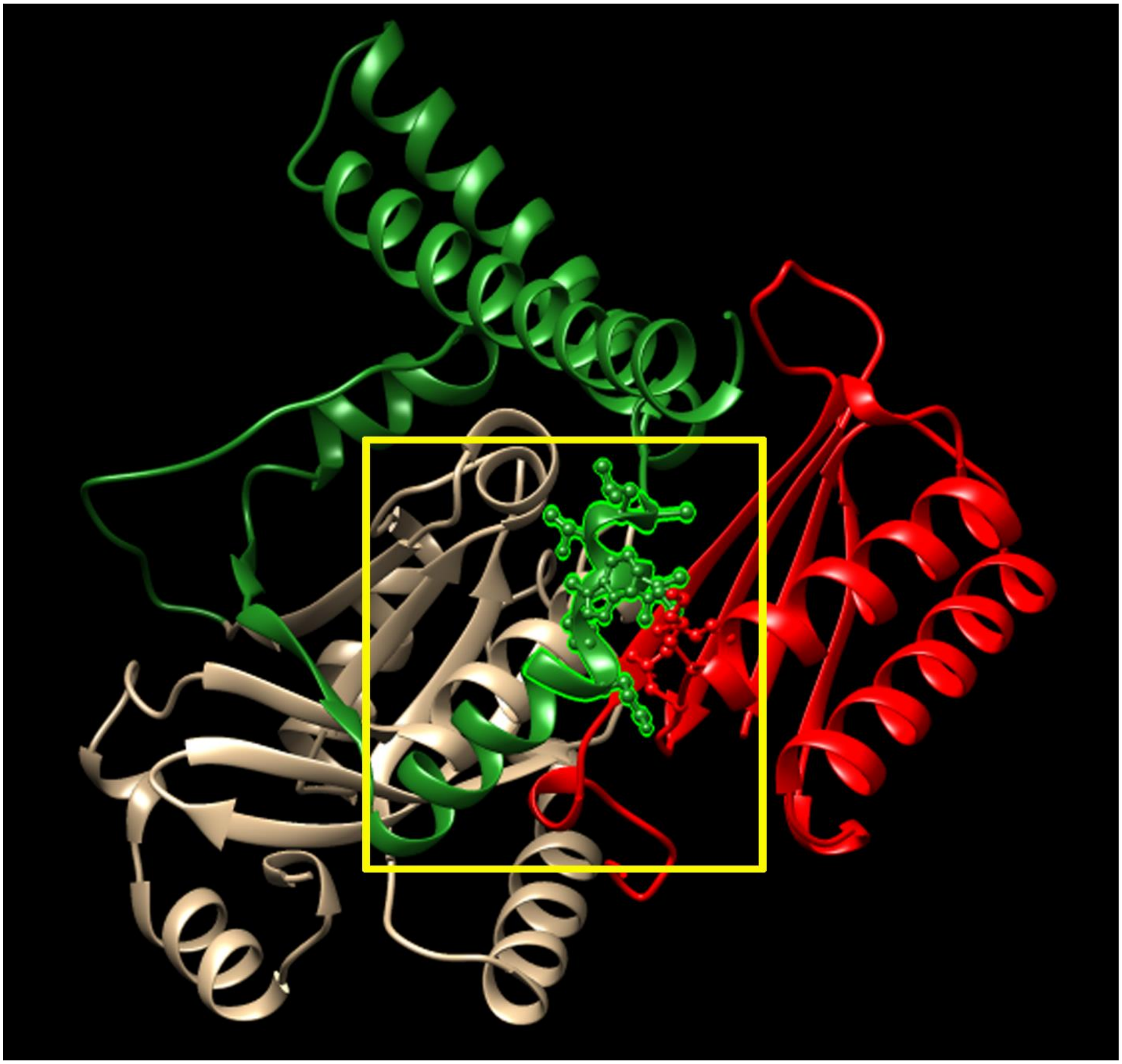

Figure.S6 Predicted *Eh*MIF-*Eh*JAB1 interaction. *Eh*MIF (red), *Eh*JAB1 C-terminal region (green) and rest of the *Eh*JAB1 protein (grey). The yellow box illustrates the C-terminal region of ∆6 *Eh*JAB1 mutant predicted to bind to *Eh*MIF, and predicted *Eh*MIF binding domain (64-69 aa) to *Eh*JAB1.

Full-length gels and blots:

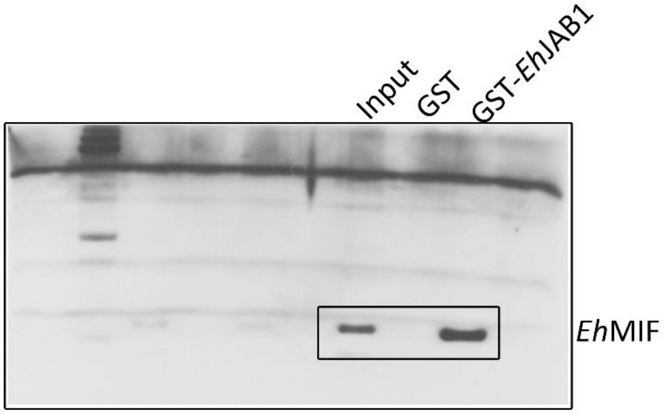

Figure 2C

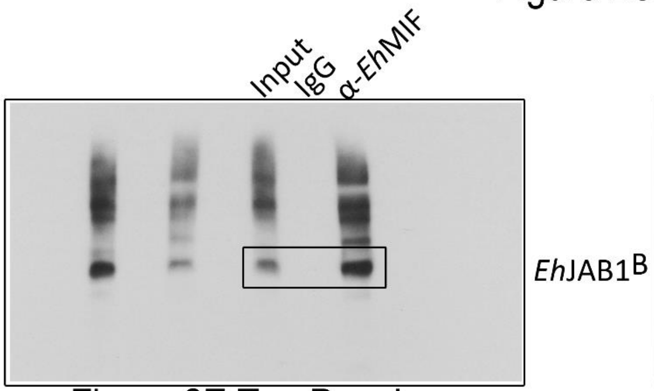

Figure 2E Top Panel

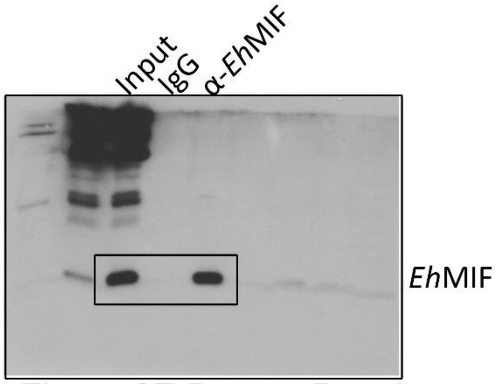

Figure 2E Bottom Panel

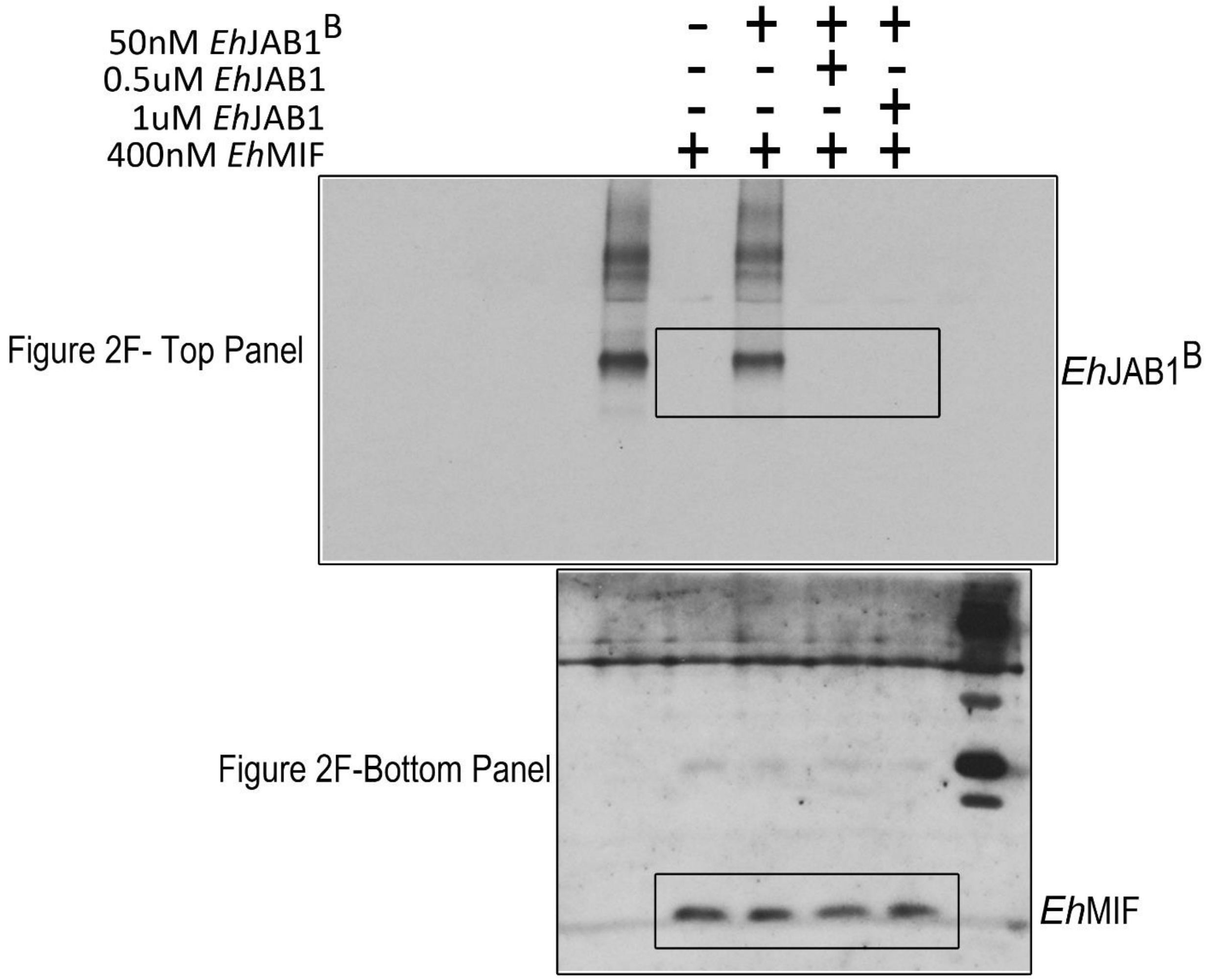
50nM EhJAB1B
0.5uM EhJAB1
1uM EhJAB1
400nM EhMIF
Figure 2F- Top Panel
EhJAB1B
Figure 2F-Bottom Panel
EhMIF

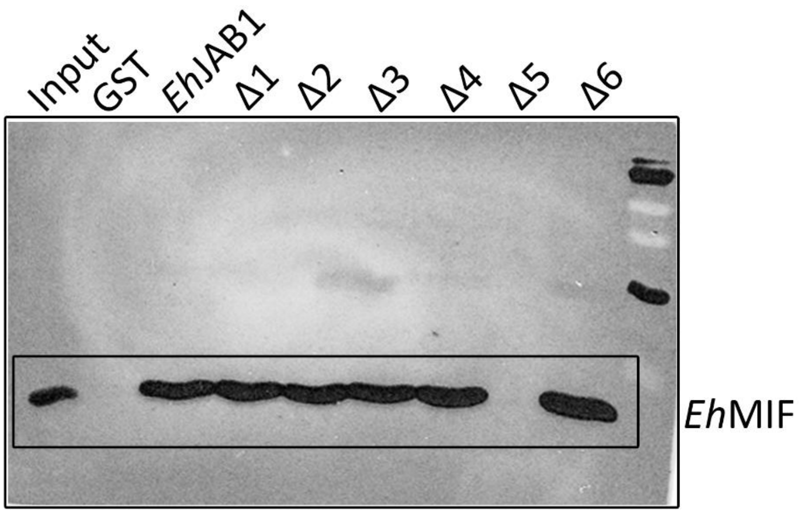

Figure 4C

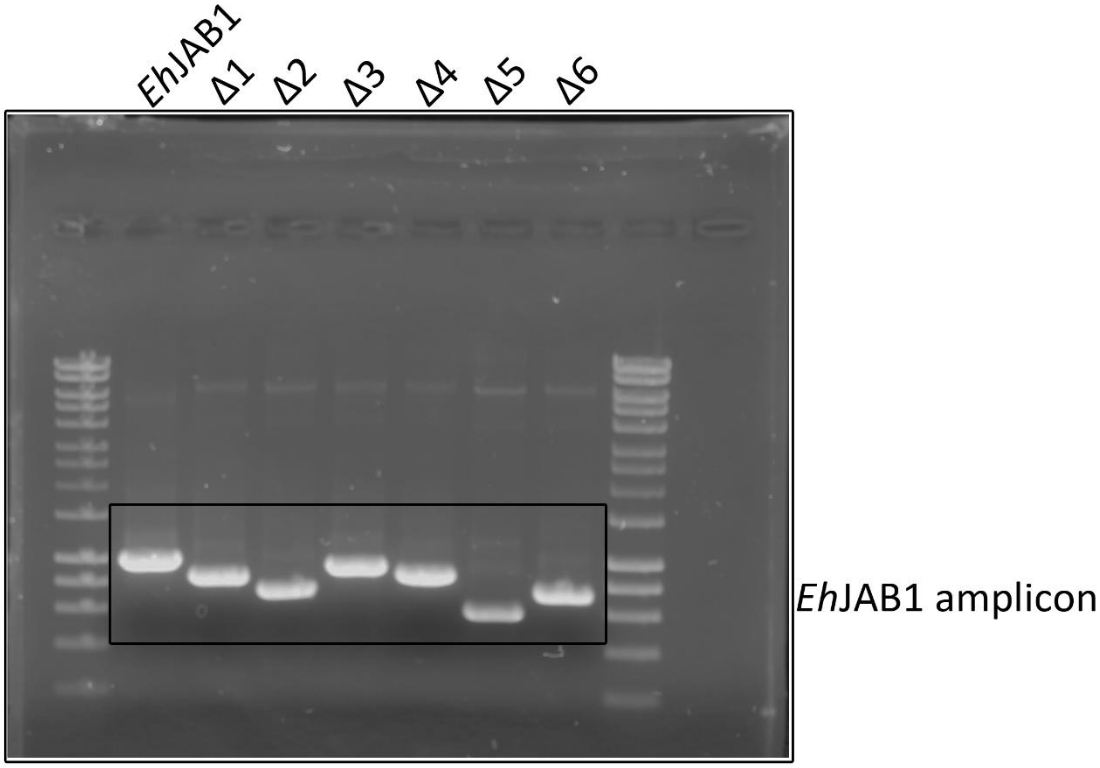

Figure 4D

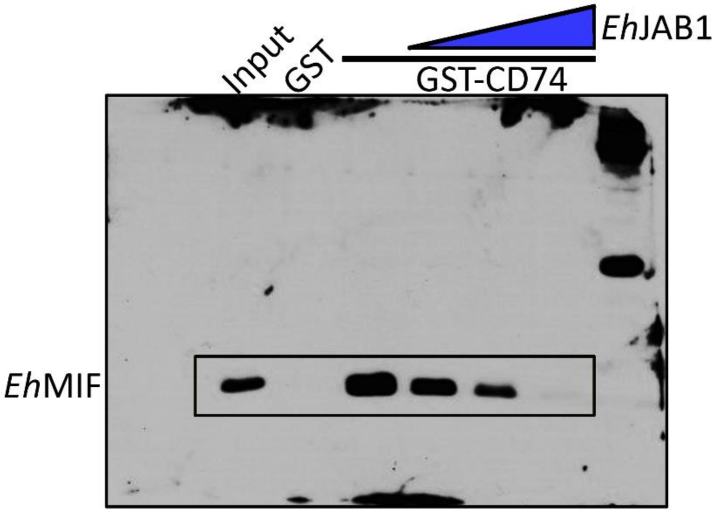

Figure 5A

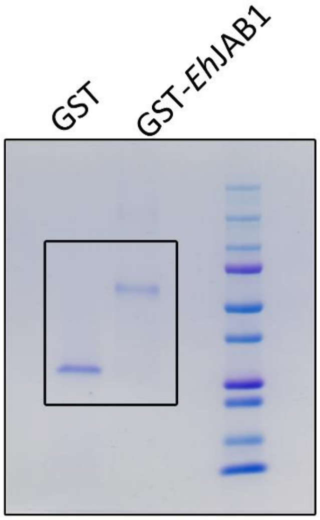

Figure 2B

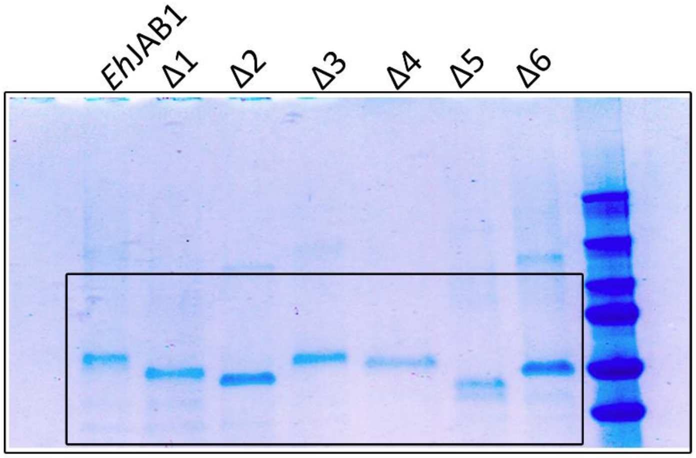

Figure 4C

Figure 3A and 3B raw data

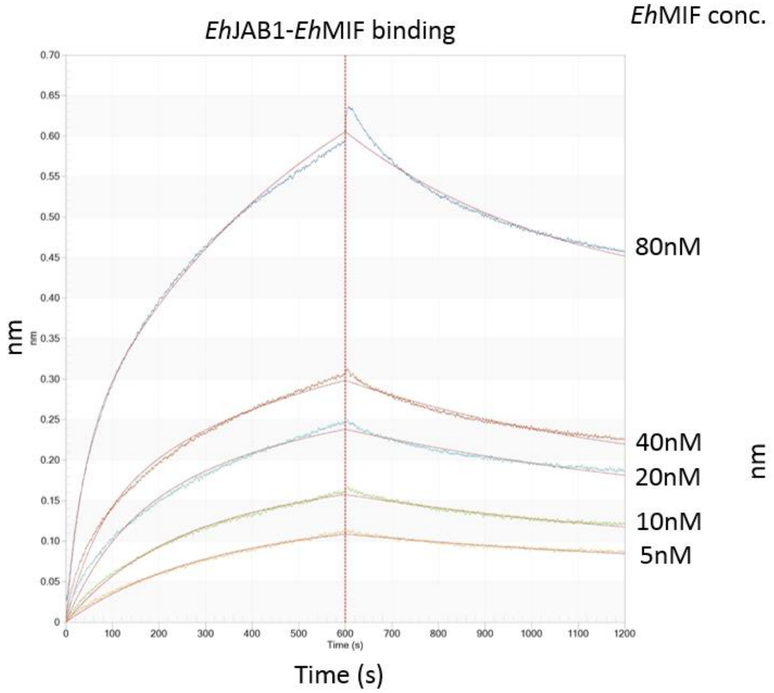

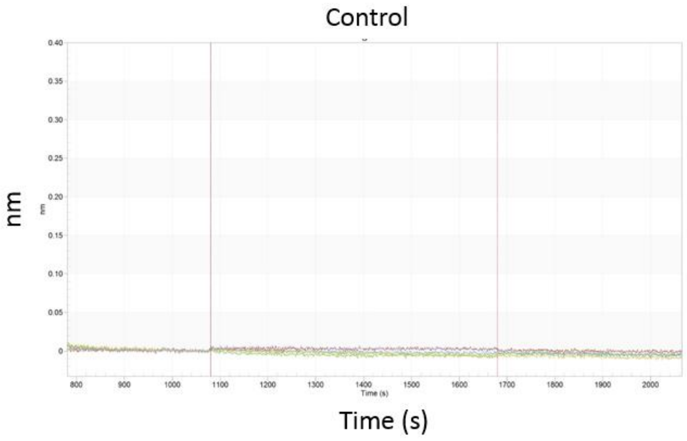

Supplement: Supplementary file 1 — Supplementary Information [file 41598_2018_28625_MOESM1_ESM.pdf]
